# Incidence Rate and Factors Associated with Delirium and Subsyndromal Delirium in Patients with COVID-19 in an Intensive Care Unit

**DOI:** 10.3390/jcm12113789

**Published:** 2023-05-31

**Authors:** Lara Helena Perpetuo, Wellington Ferreira, Danilo Jorge da Silva, Mauro Eduardo Jurno, Thiago Cardoso Vale

**Affiliations:** 1 Programa de Pós-Graduação em Saúde, Faculdade de Medicina, Associação Hospitalar Bom Jesus de Congonhas, Universidade Federal de Juiz de Fora, Congonhas 36415-000, MG, Brazil; larahcbp89@gmail.com; 2 Faculdade de Medicina, Universidade Federal de São João Del Rei, Divinópolis 35501-296, MG, Brazil; 3 Programa de Pós-Graduação em Saúde, Faculdade de Medicina, Universidade Federal de Juiz de Fora, Juiz de Fora 36036-900, MG, Brazil; 4 Faculdade de Medicina, Fhemig, Belo Horizonte 30622-020, MG, Brazil; mejurno@gmail.com

**Keywords:** delirium, COVID-19, intensive care unit

## Abstract

Background: Delirium subsyndrome (SSD) and delirium (DL) are known complications in the intensive care unit (ICU) and are associated with worse clinical outcomes. The aim of this study was to screen for SSD and DL in individuals with COVID-19 admitted to the ICU and to study the associated factors and clinical outcomes. Method: An observational, longitudinal study was conducted in the reference ICU for COVID-19. All admitted individuals with COVID-19 were screened for SSD and DL during their ICU stay using the Intensive Care Delirium Screening Checklist (ICDSC). Individuals with SSD and/or DL were compared to those without SSD and/or DL. Results: Ninety-three patients were evaluated, of which 46.7% had SSD and/or DL. The incidence rate was 4.17 cases/100 person-days. Individuals with SSD and/or DL had higher severity of illness on admission to the ICU, as measured by the APACHE II score (median 16 versus 8 points, *p* < 0.001). SSD and/or DL were associated with longer ICU and hospital stays (median 19 versus 6 days, *p* < 0.001 and median 22 versus 7 days, *p* < 0.001, respectively). Conclusion: Individuals with SSD and/or DL had greater disease severity and longer ICU and hospital stays when compared to those without SSD and/or DL. This reinforces the importance of screening for consciousness disorders in the ICU.

## 1. Introduction

The first study of the incidence of neurological manifestations in patients with COVID-19 was conducted by a Chinese group from Wuhan and found an incidence of 36.4%, being more frequent in severe cases, reaching 45.5%. Neurological manifestations, including cerebrovascular diseases, disorders of consciousness, and musculoskeletal disorders, are related to acute respiratory distress syndrome (ARDS) and are more severe in elderly and hypertensive patients [1]. In France, Helms et al. [2], in a case series of 58 cases of COVID-19 and SARS, observed psychomotor agitation when the use of neuromuscular-blocking agents was discontinued in 40 (69%) individuals. Twenty-six (65%) individuals had delirium at the screening examination.

A meta-analysis published in 2021, which included a total of 145,721 individuals in the 350 studies analyzed, indicated the association of neurological disorders and disease severity, with skeletal muscle weakness, disturbances of consciousness, and fatigue being the most common symptoms associated with severe cases. In the evaluation of the subgroup of individuals older than 60 years, which included more than 3000 cases, more than one-third (34%) had mental confusion or delirium. In this subgroup, neurological manifestations were associated with increased mortality (OR 1.80, 95% CI 1.11–2.91) [3].

Delirium syndrome (DL) is among the numerous abnormalities present during the intensive care unit (ICU) stay and is associated with an increase in morbidity and mortality and a decrease in quality of life [4,5]. As defined in the fifth edition of the *Diagnostic and Statistical Manual of Mental Disorders* (DSM-V) of the American Psychiatric Association, DL is “a disturbance of consciousness characterized by an acute onset and a fluctuating course of impaired cognitive functioning so that a patient’s ability to receive, process, store, and recall information is strikingly impaired” [6].

Subsyndromal delirium (SSD) is characterized by a change in mental status that is less intense than that of DL, including one or more symptoms of DL, without all the characteristics [7]. The incidence rate in previous studies ranged from 15.8 to 86.0%. Few studies have evaluated the incidence of conversion of a previous diagnosis of SSD to DL, which is estimated to occur in between 2.0 and 9.5% of cases [8,9,10,11,12].

Several factors contribute to the increased risk of neuropsychiatric complications in patients with COVID-19. These factors can be divided into four groups: viral factors, environmental factors, factors related to treatment, and factors related to the disease itself. An example of viral factors is the direct invasion of the CNS, which can lead to encephalopathy. Regarding the factors related to treatment, those include drugs used (antibiotics and corticosteroids), duration of mechanical ventilation (MV), deep sedation, and immobilization in bed. In addition, factors related to the disease itself include fever, dehydration, hypoxemia, and cytokine storm, while the environmental factors imposed by the pandemic include isolation and separation from the family [13].

The prevalence rate of DL in patients with COVID-19 varies between 12 and 84%. Such disparity in the rate reflects the challenges in the recognition and diagnosis of the syndrome [14]. A meta-analysis of 48 studies showed a prevalence of DL of 24.3% with an incidence of 32.4% and mortality of 44.5% [13]. The occurrence of DL increases in patients with severe manifestations of the disease, such as in the case of acute respiratory distress syndrome (ARDS), with rates of 60.0 to 85.3% for patients admitted to the ICU [2,15].

DL prevention includes nonpharmacological and pharmacological strategies. Among the nonpharmacological strategies are provision of clocks and calendars to provide temporal orientation, cognitive stimulation with friends and family visits whenever possible, early out-of-bed mobilization of patients and avoidance of physical restraints, visual and hearing aids for patients with these impairments, use of transcutaneous neuromuscular electrical stimulators, noise control (reducing alarms of infusion pumps, monitors, and conversations between employees), and ambient light control to improve quality of sleep, avoidance of problematic medications and medical complications, and management of pain [16]. All these strategies are multimodal and require structural changes in the ICUs and team involvement. Regarding pharmacological strategies, available evidence does not support the use of medications to prevent DL. However, cholinesterase inhibitors, antipsychotic agents, gabapentin and analgesics, dexmedetomidine, and melatonin have all been tried [17]. A randomized double-blind study published in 2018 evaluated the use of dexmedetomidine at low doses (from 0.2 to a maximum of 0.7 mcg/kg/h with titrations targeting a Richmond Agitation–Sedation Scale (RASS) score of −1), which proved to be effective for the prevention of DL (relative risk 0.44 and 95% confidence interval 0.23 to 0.82; *p* = 0.006) [18]. In a systematic review that evaluated the use of melatonin in an attempt to control the circadian cycle and improve sleep quality to prevent DL, there was no significant reduction in the incidence of DL in ICUs [19].

Understanding SSD and DL and the associated sociodemographic and clinical factors that could potentially lead to poor outcomes may favor their early identification and treatment, with a consequent reduction in their incidence, resulting in a shorter hospital stay combined with an improved quality of life and greater independence after discharge [13]. The objective of this study was to evaluate the incidence of SSD and DL together with their associated factors and evaluate the following clinical outcomes: duration of mechanical ventilation (MV), length of ICU stay, length of hospital stay, and mortality.

## 2. Materials and Methods

### 2.1. Design

This study is characterized as quantitative, observational, longitudinal research, carried out through the collection and analysis of data from patients hospitalized in an ICU study location.

### 2.2. Study Location

The study was conducted in the ICU of Associação Hospitalar Bom Jesus (AHBJ), located in the city of Congonhas, MG, Brazil. Data collection occurred for a period of one year (20 October 2020 to 20 October 2021) and was performed twice a day (08:00 h and 20:00 h). Nurses on duty, previously trained to apply the Intensive Care Delirium Screening Checklist (ICDSC), RASS, and Behavioral Pain Scale (BPS) scales, collected the data during the day and night shifts.

### 2.3. Assessment Instruments

The ICDSC [20], which was developed based on the DL diagnostic criteria of the fourth edition of the DSM, was used for screening [21]. The scale consists of the evaluation of eight variables: attention/orientation, hallucinations, level of consciousness, psychomotor agitation, speech and mood disorder, sleep–wake cycle, and symptom fluctuation. Each item has a value of 1 point, and the total score can range from 0 to 8 points, with a cutoff point greater than or equal to 4 for the diagnosis of DL. Scores between 1 and 3 are classified as SSD. The RASS and BPS were also applied to assess each patient’s level of consciousness and/or sedation and the presence of pain, respectively [22]. The scales were validated for the Brazilian population in 2008 [23] and 2017 [24], respectively.

### 2.4. Sample

A total of 632 patients were admitted to the hospital and had later a confirmed diagnosis of COVID-19 during the period of the study. Out of 632, 135 individuals (21.4%) were screened at the ICU and 93 (14.7%) fulfilled the inclusion criteria for the study. The inclusion criteria were individuals admitted to the ICU with suspected COVID-19 and later confirmed by RT-PCR for SARS-CoV-2; age > 18 years; and agreement of a family member or guardian of the patient to participate in the study. The exclusion criteria were individuals suspected of COVID-19 not confirmed by RT–PCR for SARS-CoV-2; individuals without complete data in the screening scales; presence of coma or deep sedation with a score of −4 or −5 on the RASS scale; and patients unable to complete the scales (severe dementia).

The study sample was divided into two groups: individuals diagnosed with COVID-19 who did not have SSD and/or DL, and individuals with COVID-19 who had SSD and/or DL at some point during ICU admission. Once included in the study, the patients were followed until the final outcome: death or hospital discharge.

### 2.5. Data Collection

To analyze and establish correlations between the factors associated with the development of SSD and DL, several variables were defined, such as sociodemographic characteristics; severity of the clinical condition at the time of admission to the ICU; and laboratory tests and clinical outcomes that have already been shown to be determinants for the studied conditions [8,25]. The collected data were organized into a specific questionnaire. It was applied within 24 h of admission to the ICU.

### 2.6. Statistical Methods

The incidence rate is expressed in cases/100 person-days and was calculated by dividing the new cases by the sum of persons-time at risk multiplied by 100. All continuous variables are presented as medians and interquartile ranges (IQRs), and the frequencies of the categorical variables were calculated by direct counting and as percentages. Comparisons between continuous variables were performed using the Mann–Whitney test or the Wilcoxon signed rank test for independent and paired variables, respectively. The association between categorical variables was tested using the chi-square or Fisher’s exact method. The critical *p* value for rejecting the null hypotheses was 0.05. All analyses were performed using SPSS statistics software, version 22.0.

### 2.7. Ethical Considerations

The study was approved by the Ethics Committee of the University Hospital of UFJF under number CAAE: 30752120.0.0000.5133. Those responsible for the patients received and signed an informed consent form upon admission of the patient to the ICU.

## 3. Results

The initial study population consisted of 210 patients. Of these, 38 (18.1%) were not screened using the ICDSC scale, either due to a stay of less than 24 h or due to inapplicability of the scale, thus resulting in sample loss. Therefore, 172 patients were screened for SSD and/or DL during hospitalization. After obtaining the RT–PCR results for SARS-CoV-2, 37 screened patients were excluded from the study because they were negative for SARS-CoV-2.

**Figure 1:**
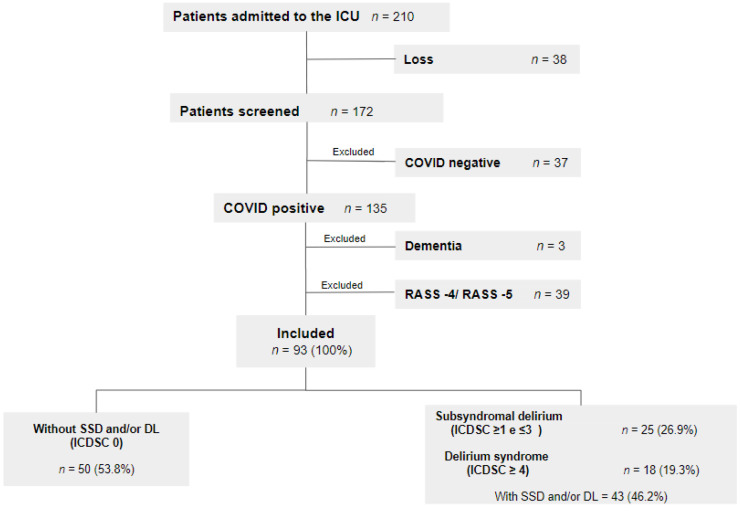
Population diagram with eligibility criteria. Abbreviations: SSD, subsyndromal delirium; DL, delirium; ICDSC, Intensive Care Delirium Screening Checklist; RASS, Richmond Agitation–Sedation Scale. ICU, Intensive Care Unit.

Of the 135 patients positive for COVID-19, 42 were excluded from the study: 3 due to previously diagnosed dementia and 39 because they were using neuromuscular-blocking agents or had a RASS score equal to −4 or −5 until death, making it impossible to detect signs of SSD and/or DL. A total of 93 patients were evaluated, of whom 25 (26.9%) had a diagnosis of SSD, 18 (19.3%) had DL, and 50 (53.8%) did not have SSD and/or DL (Figure 1). A conversion from SSD to DL was observed in 8 patients.

Individuals in the group diagnosed with SSD and/or DL had a median age of 62 years with an IQR of 23 years. For the group without SSD and/or DL, the median age was 55 years (IQR 27), *p* = 0.06. Other aspects of the population profile are described in Table 1.

**Figure 2:**
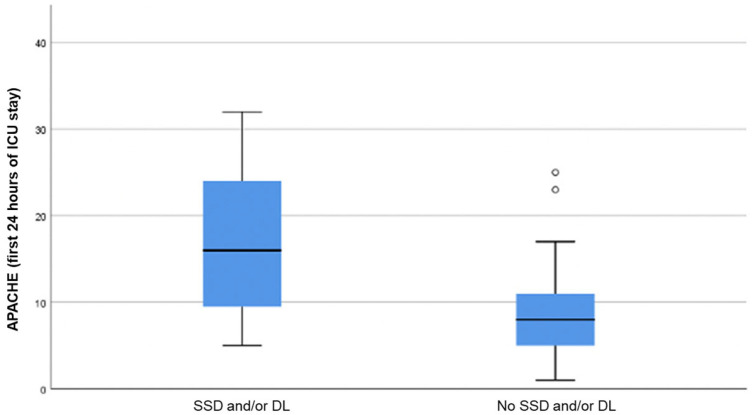
Score on the APACHE II scale in the first 24 h of admission to the ICU. SSD: subsyndromal delirium; DL: delirium; ICU: intensive care unit; APACHE: Acute Physiology and Chronic Health Evaluation-II.

The incidence rate of SSD and/or DL was 4.17 cases/100 person/day (4.17%). It took a median of 6.0 ± 14.0 (3.5–17.0) days after hospital admission for the development of SSD and/or DL. The median Acute Physiology and Chronic Health Evaluation-II (APACHE II) score in the first 24 h of hospitalization for patients with SSD and/or DL was 16.0 ± 15.0 (9.0–24.0) points, significantly higher than the score for patients without SSD and/or DL, with a median of 8.0 ± 6.0 (5.0–11.0) points, *p* < 0.001 (Figure 2). Eight patients (18.6%) had tracheostomy when SSD/DL was diagnosed. These patients took a mean of 17.0 ± 5.0 days (15–20) from the ICU admission until tracheostomy and remained with the tracheostomy for a mean of 16.0 ± 5.0 days (14.5–19.5).

Laboratory tests were conducted on the day that the individuals presented SSD and/or DL and on the day of admission for patients in the control group. Partial pressure of carbon dioxide (pCO_2_), sodium, potassium, hemoglobin, and urea were significantly different between the two groups. In contrast, the other laboratory parameters, namely lactate, serum magnesium, hydrogenic potential (pH), creatinine, and C-reactive protein, were not significantly different between the study populations, as detailed in Table 2. Thirty-three (76.7%) patients with SSD and/or DL used a benzodiazepine at some point during hospitalization. We did not observe a significant association between the development of SSD and/or DL with the use of benzodiazepine in our sample (*p* = 0.309). None of our patients had physical restraints prior to the development of SSD and/or DL.

The outcomes analyzed were length of ICU stay, length of hospital stay, and duration of MV (Table 3). The length of ICU stay and hospital stay were significantly longer in the group with SSD and/or DL. Thirty-one (72.1%) patients with SSD and/or DL and thirteen (26.0%) patients without SSD and/or DL were on MV. The risk for development of SSD and/or DL when placed on MV was 2.88 times higher when compared to those not placed on MV (*p* < 0.001; CI 95% 1.70–4.88). The duration of MV was not different between the sample and control groups (*p* = 0.847). Regarding mortality, of the 92 patients evaluated (43 who developed SSD and/or DL and 49 without SSD and/or DL), there was no significant difference between the sample and control groups, with 11 and 10 deaths, respectively.

## 4. Discussion

In this study, individuals with COVID-19 with SSD and/or DL were compared with those without SSD and/or DL, and there was no statistically significant difference regarding age, education level, and comorbidities analyzed. The incidence rate of SSD and/or DL was 4.17 cases/100 person-day (4.17%). The APACHE II score was used to assess the severity of the individuals admitted to the ICU, and there was a significant association between SSD and/or DL and disease severity. The abnormal laboratory tests that were relevant in the comparison between the groups were pCO_2_, Hb, and urea. Regarding the clinical outcomes studied, there was no statistical significance in the duration of MV and mortality, but the length of ICU stay and hospital stay were longer in individuals who presented cognitive changes. Data were collected mostly during the second wave of the COVID-19 pandemic in Brazil, which lasted until 30 April 2021. The Brazilian hospital system suffered from a scarcity of resources, especially related to drugs such as analgesics, sedatives, and neuromuscular blockers, which had to be substituted to second-line agents.

The ICDSC has a sensitivity of 99% and specificity of 64%, with an area under the ROC (receiver operating characteristic) curve of 0.9 [20]. The ICDSC scale was validated for Portuguese with a sensitivity of 96.0%, specificity of 72.4%, and a high accuracy based on the DSM-IV criteria, with an area under the ROC curve of 0.91 [26]. The scale can be quickly applied, lasting around one to two minutes, appropriate for SSD and/or DL screening in ICUs by previously trained physicians, nurses, and residents [20,22,27,28], especially during the pandemic, when the team was submitted to a high workload.

Few cohort studies have evaluated DL only in critically ill patients with COVID-19 admitted to ICUs. The incidence of DL in this population of critically ill individuals ranged from 51.4% to 84.0% [2,15,29,30]. The literature lacks reports of SSD in COVID-19. In a Japanese study performed in general (medical and surgical) ICUs involving 380 hospitalized individuals followed for six months, the incidence of SSD was 33.9%, the incidence of DL was 15.8%, and the conversion of SSD to DL was 9.5% [8]. Another Canadian cohort involving 537 individuals admitted to general ICUs for one year showed the presence of SSD and DL in 33.3% and 35.2% of cases, respectively [8].

Regarding the prevalence of DL in patients with COVID-19, a retrospective cohort study reported DL as the sixth most common sign or symptom of COVID-19. In addition, advanced age (>75 years) was reported as the main risk factor for the development of DL during hospitalization due to SARS-CoV-2 infection. Another retrospective study of the prevalence of DL in elderly patients with COVID-19 showed that the median age was 86 years, indicating advanced age as a factor correlated with the onset of DL during COVID-19. In our study, the median age of patients diagnosed with SSD and/or DL was 62 years (IQR 23 years), which was not significantly different from the group without SSD and/or DL, with a median of 55 years (IQR 27 years); therefore, age did not affect the onset of SSD and/or DL in our study. The absence of this association contradicts some studies that indicate a higher incidence of SSD in older populations, with an age over 65 years as an important risk factor for the onset of the condition. Fong et al. highlighted that age over 65 years is a risk factor for DL and that the incidence may increase to 70–87% in patients admitted to the ICU [31]. In addition, in a more recent study by Hshieh et al., age above 75 years was established as a risk factor for the development of DL, with an increase in DL in this age group mainly due to greater longevity of the population, better quality of life, and advances in palliative treatments [32]. A possible explanation for the development of SSD and/or DL in younger patients (<65 years) in our study is the multifactorial nature: including the association of previous comorbidities, severity of the condition, and diagnosis of COVID-19, a syndrome that, based on several mechanisms mentioned later, may be the cause of SSD and/or DL.

Our study showed that individuals who presented with DL during the ICU stay were admitted with more severe clinical manifestations of the disease. The association of DL with disease severity has been described in studies with different populations, including people with COVID-19 [29,33,34,35,36,37]. Among the evaluated clinical outcomes in our study, the length of ICU stay was longer for patients with SSD and/or DL, as was the length of hospital stay, which increased more than three-fold. These findings corroborate the evidence presented by other studies [29,30,36,38] such as that performed by Khan et al., a retrospective study that included 268 patients with COVID-19, of whom 215 (80.2%) had DL and a longer ICU stay [36].

Regarding mortality, there was no significant difference between the groups in our study. A cohort study conducted by Williamson et al., who studied DL in 213 ICU patients with COVID-19, found that DL was associated with longer duration of MV, longer ICU stay, and worsening of functionality; however, there was no difference in mortality between the populations with and without DL (*p* = 0.17) [29]. An Italian study that performed a retrospective analysis of hospitalized individuals with COVID-19 who presented DL found, in the univariate analysis, that mortality worsened (57 versus 30%; *p* < 0.001); however, in the multivariate regression analysis, there were confounding factors such as respiratory disease, which did not allow DL to be considered an independent predictor of mortality [37]. In the multicenter study by Pun et al., cited above, of the 2088 individuals studied, 601 (28.8%) died within 28 days, and most of the deaths occurred in the ICU [35]. There are studies that present mortality as an independent risk for DL, but the population of these studies is mostly hospitalized patients, including in wards and ICUs [3,38]. Our study population was recruited from the ICU, where individuals have the most severe manifestations of the disease, and this may have contributed to the lack of a significant difference.

One of the limitations of our study is the fact that the cohort was obtained from a single health center with only 10 ICU beds; therefore, the findings cannot be generalized to other institutions in the state or country. The sample was biased because it was exclusively selected from the ICU, where patients with more severe manifestations of COVID-19, especially ARDS, are found. In addition, we did not exclude patients in a state of shock, which could have also impaired the mental status. Assessment of capillary refill time could have been an easy and rapid tool [39] to detect hypoperfusion in our sample, instead of the lactate level [40]. We used a specific questionnaire, not externally validated prospectively, to analyze the factors associated with the development of SSD and/or DL, which reduced the precision of the data collected.

## 5. Conclusions

In this study, the incidence rate of SSD and DL in individuals with COVID-19 admitted to the ICU of Congonhas from October 2020 to October 2021 was 4.17 cases/100 person-days. SSD and DL were associated with the severity of COVID-19 in individuals at the time of admission to the ICU, longer ICU and hospital stays, and there was no association with MV time and mortality. In the pandemic scenario, screening for SSD and DL is important to initiate early treatment, reduce hospital costs, and increase bed turnover.

## Figures and Tables

**Table 1:** Population profile of the group of patients with subsyndromal delirium and/or delirium and the group of patients without subsyndromal delirium and/or delirium.

Variables	Categories	SSD and/or DL			No SSD and/or DL			*p*
		n	%	25th–75th	n	%	25th–75th	
Sex	Male	27	62.8		37	74		0.250 *
	Feminine	16	37.2		13	26		
Age		62 (23) ^a^		52–35	55 (27) ^a^		42.5–69.5	0.055 **
	75 years or less	10	23.3		8	16		0.377 *
	over 75 years	33	76.7		42	84		
Education level	Illiterate	7	16.3		4	8		0.200 *
	Complete elementary education	15	34.9		15	30		
	Incomplete elementary education	8	18.6		11	22		
	Complete high school	9	20.9		19	38		
	Incomplete high school	4	9.3		1	2		
Systemic arterial hypertension	Yes	20	46.5		19	38.0		0.407 *
Diabetes mellitus	Yes	18	41.9		13	74.0		0.106 *

Abbreviations: SSD, subsyndromal delirium; DL, delirium. ^a^ Median values (IQR). * *p*-value for x^2^ or Fischer’s exact tests. ** *p*-value for Mann–Whitney’s test. *p* considered significant < 0.05.

**Table 2:** Difference in laboratory variables between the group of patients with subsyndromal delirium and/or delirium and the group of patients without subsyndromal delirium and/or delirium.

Variables	SSD and/or DL		No SSD and/or DL		*p* *
	Median (IQR)	25th–75th	Median (IQR)	25th–75th	
pH	7.4 (0.1)	7.4–7.5	7.4 (0.1)	7.4–7.5	0.228
pCO2 (mmHg)	41.5 (12.0)	36.0–48.0	37.5 (8.0)	33.0–41.0	0.028
Lactate (mg/dL)	14.0 (4.8)	10.2–15.0	12.5 (7.0)	10.0–17.0	0.801
Hemoglobin (g/dL)	11.1 (4.2)	8.5–12.7	13.1 (2.1)	12.4–14.5	<0.001
Urea (mg/dL)	69.5 (44.5)	44.7–89.2	46.5 (27.5)	31.8–59.3	0.002
Creatinine (mg/dL)	1.0 (0.7)	0.8–1.5	0.9 (0.3)	0.8–1.1	0.326
Sodium (mEq/L)	137.0 (7.5)	133.8–141.3	135.0 (4.0)	133.0–137.0	0.027
Potassium (mEq/L)	3.8 (0.8)	3.3–4.1	4.1 (1.1)	3.6–4.7	0.017
Magnesium (mg/dL)	1.8 (0.4)	1.6–2.0	1.9 (0.4)	1.7–2.1	0.651
CRP (mg/L)	76.7 (38.3)	56.8–95.1	83.5 (71.7)	43.6–115.3	0.449

Abbreviations: SSD, subsyndromal delirium; DL, delirium; pH, hydrogenic potential; pCO2, partial pressure of carbon dioxide; CRP, C-reative protein; IQR, interquartile range. * *p*-value for Mann–Whitney’s test. *p* considered significant < 0.0.5.

**Table 3:** Clinical oucomes: length of ICU stay, length of hospital stay, duration of mechanical ventilation and mortality between groups.

Variables	SSD and/or DL		No SSD and/or DL		*p*
	Median (IQR) or n	25th–75th	Median (IQR) or n	25th–75th	
Length of ICU stay (days)	19.0 (28)	10.0–38.0	6.0 (7.7)	3.8–11.5	<0.001 *
Length of hospital stay (days)	22.0 (27)	13.5–40.5	7.0 (6)	6.0–12.0	<0.001 *
Duration of mechanical ventilation (days)	14.0 (21)	7.0–28.0	14.0 (13.5)	9.5–23.0	0.847 *
Mortality	11 ^a^		10 ^a^		0.555 **

Abbreviations: SSD, subsyndromal delirium; DL, delirium; IQR, interquartile range. ^a^ absolute values. * *p*-value for Mann–Whitney’s test. ** *p*-value for x^2^ test. *p* considered significant < 0.05. ICU: intensive care unit

## Data Availability

The datasets used and analyzed during the current study are available from the corresponding author on reasonable request.
